# Skin-Resident Antigen-Presenting Cells: Instruction Manual for Vaccine Development

**DOI:** 10.3389/fimmu.2013.00157

**Published:** 2013-06-20

**Authors:** Cynthia M. Fehres, Juan J. Garcia-Vallejo, Wendy W. J. Unger, Yvette van Kooyk

**Affiliations:** ^1^Department of Molecular Cell Biology and Immunology, VU University Medical Center, Amsterdam, Netherlands

**Keywords:** skin, antigen-presenting cells, vaccination, microneedles, C-type lectin receptors, glycans

## Abstract

The induction of antigen-specific effector T cells is driven by proper antigen presentation and co-stimulation by dendritic cells (DCs). For this reason strategies have been developed to instruct DCs for the induction of CD4^+^ and CD8^+^ T cell responses. Since DCs are localized, amongst other locations, in peripheral tissues such as the skin, new vaccines are aiming at targeting antigens to DCs *in situ*. Optimal skin-DC targeting in combination with adequate adjuvant delivery facilitates DC maturation and migration to draining lymph nodes and enhances antigen cross-presentation and T cell priming. In this review we describe what DC subsets populate the human skin, as well as current vaccination strategies based on targeting strategies and alternative administration for the induction of robust long-lived anti-cancer effector T cells.

## Introduction

Immature dendritic cells (DCs) patrol the tissues to sense for pathogens. Recognition of pathogens through specific innate receptors allows antigen uptake and processing for presentation on MHC class I and II molecules, while DCs migrate to secondary lymphoid organs. Upon entry in the lymph nodes, DCs display a mature phenotype, characterized by high expression levels of co-stimulatory molecules and the production of pro-inflammatory cytokines. The presentation of antigenic peptides on MHC class I or II molecules combined with the signals derived from mature DCs allows the initiation of antigen-specific humoral and cellular immune responses (Ueno et al., 2007). Besides triggering activating immune responses aiming at the clearance of pathogens, DCs are also capable to down-modulate unwanted auto-immune reactions by maintaining immune tolerance via the induction of regulatory T cells (Maldonado and von Andrian, 2010). Consequently, as DCs are able to induce both activating as well as suppressive immune responses, this makes them ideal targets for vaccination strategies against cancer or autoimmune disorders.

During the past years, extensive research has focused on the development of DC-targeting vaccination strategies against cancer. First attempts focused on pulsing DCs *ex vivo* with tumor antigens and maturation agents (Tacken et al., 2007). This strategy involved the generation of DCs from monocytes or CD34^+^ precursors and the subsequent re-injection into the patient to generate effective T and B cell responses against the tumor. Although this system is well tolerated by the patients and have shown modest clinical responses (Galluzzi et al., 2012), there are limitations to *ex vivo* culture and antigen-loading of DCs. Firstly, it has been shown that *ex vivo* cultured DCs exhibited reduced migratory potential and secondly, the development of personalized vaccines is costly and difficult to standardize. By comparison, higher objective clinical response rates have been obtained by immunotherapies based on adoptive transfer of tumor-specific T cells [either *ex vivo* expanded tumor infiltrating lymphocytes (TILs) or T cells transduced with high affinity TAA-specific TCR or chimeric antigen receptors (CARs)] (Gattinoni et al., 2012; Restifo et al., 2012; Turtle et al., 2012). The success of this type of immunotherapy is shown to depend on the number and differentiation status of adoptively transferred T cells (Gattinoni et al., 2005; Klebanoff et al., 2011). Additionally, several studies point to improved anti-tumor efficacy when the T cells are activated immediately prior to or directly after adoptive transfer. The latter could be accomplished by co-administration of a tumor-antigen vaccine (Overwijk et al., 2003; de Witte et al., 2008a,b; Klebanoff et al., 2009). However, also this therapy is very costly and since effective TILs seem to be restricted to melanoma and genetically engineered T cells only possess monoclonal specificity, targeting DCs directly *in vivo* therefore provides an attractive alternative.

The goal of *in vivo* DC-targeting vaccines is twofold, accumulating antigens to DCs in a cell-specific manner while promoting antigen uptake, cross-presentation, and DC maturation. In order to achieve this, knowledge on the selective expression of antigen-uptake receptors on various DC subsets is required, as well as which DC subsets has superior cross-presentation capacity. DCs express a multitude of pattern recognition receptors, such as Toll-like receptors (TLRs) and C-type lectins (CLRs). While TLRs play a crucial role in pathogen recognition, the induction of DC maturation, and the production of inflammatory cytokines, CLRs have been shown to have a subset-specific expression pattern and are able to mediate antigen uptake and cross-presentation. Already more than 10 years ago, work from the group of Steinman showed that the CLR DEC-205 mediated the uptake of ovalbumin coupled to a DEC-205 antibody, resulting in increased CD4^+^ and CD8^+^ T cell activation (Bonifaz et al., 2002, 2004). Since then, the targeting of several CLRs with antigens conjugated to monoclonal antibodies (mAbs), including mAbs against mannose receptor (MR), CLEC9A, DC-SIGN, and Langerin, has been explored (Caminschi and Shortman, 2012). While some of these CLRs are not considered DC specific, such as DEC-205 and MR, others such as DC-SIGN, CLEC9A, and Langerin are expressed on specific DC subsets [myeloid DCs (mDC), plasmacytoid DCs (pDCs), and Langerhans’ cells, respectively]. Although several studies in search of the most efficient cross-presenting DC subset have demonstrated the CLEC9A^+^ pDC as the most potent one in cross-presenting soluble antigen compared to LC and mDC, it may still be that for certain glycosylated antigens that target Langerin^+^ LC and DC-SIGN^+^ mDC, these subsets might have similar potential as the CLEC9A^+^ pDC to cross-present (Tel et al., 2012, 2013; Unger et al., 2012). In general, it has become clear that only the simultaneous delivery of CLR-targeting antigens together with a potent adjuvant will lead to the generation of efficient CD4^+^ and CD8^+^ T cell responses, especially in the context of anti-tumor immune therapies.

In addition, DC subsets present in the various tissues and lymphoid organs do not all express the same level and variety of CLRs and not all DC subsets are equally potent in activating CD4^+^ and CD8^+^ T cells. By deliberate selection of the right DC subset and/or through targeting of CLRs specifically expressed by DC subsets, an optimal induction of cellular immune responses (either immunity or tolerance) can be achieved. Although a lot of data has been accumulated on DC targeting strategies in *in vivo* systems in mice, still little is known on the efficacy of DC-targeting vaccines in human skin. Here, we review recent knowledge on DC subsets residing in the human skin, including their CLR and TLR expression, capacity to cross-present antigens, and to respond to adjuvants for migration to draining lymph nodes. Finally, new developments on the strategies used to selectively deliver vaccines to specific layers within the skin, as well as how to overcome potential side effects of the immune suppressive skin micromilieu will be discussed.

## DC Subsets: Differences in Function and CLR and TLR Expression

**Figure 1:**
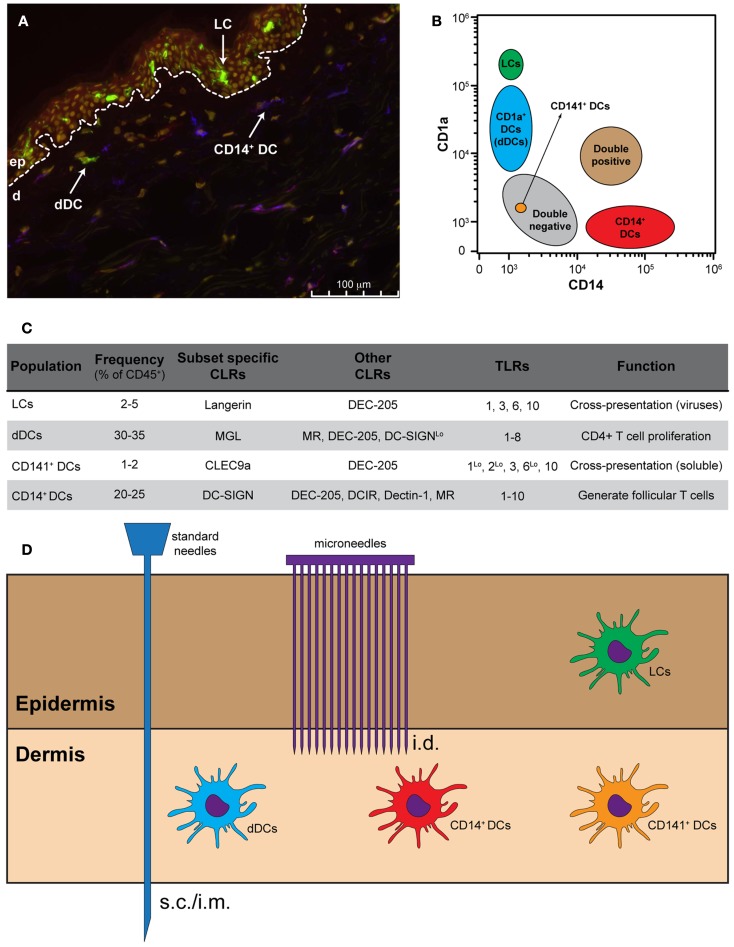
**Targeting skin APCs**. **(A)** Representative immunofluorescence staining of human skin using monoclonal antibodies against CD1a (green), CD14 (red), and DC-SIGN (blue). Nuclei are stained with the nuclear dye Hoechst (yellow). **(B)** Distribution of skin APCs according to their CD1a and CD14 expression levels. **(C)** Frequency, CLR and TLR expression and function of the four main skin APC subsets. **(D)** Standard needles do not allow skin APC targeting, while different models of microneedles allow for the specific targeting of dermal APCs and, in some cases, also LCs.

The human skin has been classically divided in two main compartments: the epidermis and the dermis. The epidermis is the outer layer that provides the barrier function to the skin. Within the epidermis frequently dividing keratinocytes are located, melanocytes, which produce the pigment melanin, and LCs, which are the main epidermis-resident antigen-presenting cell (APC) and are characterized by the expression of the CLR Langerin (Figure 1). In addition, T cells, mainly CD8^+^ T cells, can be found in the epidermis. Whereas the epidermis has a relatively simple histology, the underlying dermis is anatomically more complex and accumulates greater cell diversity. The dermis is rich in many specialized immune cells, including dermal DCs, CD4^+^ T helper (Th) cells, γδ T cells, and natural killer T (NKT) cells. Moreover, macrophages, mast cells, fibroblasts, and nerve-related cell types are also present. The dermis is drained by lymphatic and vascular conduits, through which migrating cells can traffic.

In both human and mice, two main lineages of skin-resident DCs are known: pDCs and the tissue-resident mDC. Steady-state human skin contains four phenotypically and functionally distinct subsets of DCs. Within the epidermis CD1a^*high*^ LCs can be found, whereas the CD1a^+^, CD14^+^, and CD141^+^ DCs (also known as BDCA3^+^ DCs) are present within the dermis (Klechevsky et al., 2008, 2009; Haniffa et al., 2012; Segura et al., 2012). The latter subset has recently been described in the dermis of human skin as a rather efficient DC in the cross-presentation of antigen (Haniffa et al., 2012). In addition, high expression levels of CCR7 coincided with superior migratory behavior of these DC (van de Ven et al., 2011; Haniffa et al., 2012). Furthermore, LCs have also been described as very efficient in the priming and cross-priming of CD8^+^ T cells, whereas CD14^+^ dermal DCs (dDCs) are able to induce the generation of follicular Th cells (Klechevsky et al., 2008; Banchereau et al., 2012). The precise function of the CD1a^+^ dDCs is still poorly defined, although it has been shown that they are capable of stimulating CD4^+^ T cell proliferation (Klechevsky et al., 2009). It has also been shown that LCs are less responsive to bacteria due to a lack of TLR2, TLR4, and TLR5 expression, but are known to express TLR1, TLR3, TLR6, and TLR10, making them suitable to respond to viruses (Angel et al., 2007; van der Aar et al., 2007; Klechevsky et al., 2009). CD14^+^ dDCs express most of the 10 human TLRs, while CD1a^+^ dDCs seem to express all TLRs with exception of TLR9 and TLR10 (Angel et al., 2007; van der Aar et al., 2007). In contrast, the CD141^+^ DC expresses high levels of TLR3 and TLR10 and moderate expression of TLR1, TLR2, and TLR6 (Hémont et al., 2013). In response to polyI:C, this subset produces inflammatory cytokines such as TNF-α and IL-1β (Haniffa et al., 2012).

The four skin resident DC subsets can also be separated based on their expression pattern of CLRs: the CD14^+^ dDCs typically express DC-SIGN, DEC-205, DCIR, Dectin-1, and MR (Klechevsky et al., 2009), whereas the CD1a^+^ dDCs can be distinguished based on the expression of MGL and low levels of MR, DEC-205, and DC-SIGN (Unger and van Kooyk, 2011) (Figure 1). In humans, LCs are the only cells that express Langerin. Additionally, LCs also express DEC-205 at intermediate levels. In contrast, the CD141^+^ DC express the CLRs DEC-205 and CLEC9A, which is described to be involved in the uptake of dead cells (Poulin et al., 2010; Meixlsperger et al., 2013). Therefore, LCs could be exclusively targeted via Langerin, whereas specific targeting of CD14^+^ dDCs should be possible via DC-SIGN.

## Targeting Antigens to DC Subsets through Specific C-Type Lectins

C-type lectins such as DEC-205 and CLEC9A have shown their capacity to internalize antigen for presentation to CD4^+^ T cells and cross-presentation for the induction of antigen specific CD8^+^ T cells. Often antibodies specific for these receptors have been used for targeting purposes (Bonifaz et al., 2004; Caminschi et al., 2008). In contrast, Langerin, MR, and DC-SIGN can be either targeted using specific mAbs or using their natural ligands, since for these receptors the glycan-binding profile has been determined (Holla and Skerra, 2011; Lee et al., 2011; Unger et al., 2012). The use of glycans reports several advantages, such as the relatively easy production of glycans in large scale and their lower immunogenicity as compared to mAbs. The potential use of glycan-based DC-targeting vaccines applied via the skin has hardly been investigated. Often the cross-presenting capacity of skin-resident DC subsets has been investigated, by analyzing the potency of DC subset to cross-present soluble antigen. In these studies the CD141^+^ DC subsets have shown to have a superior cross-presenting capacity, whereas LCs, CD14^+^, and CD1a^+^ DCs do not have this potential. Conjugation of antigen to anti-Langerin antibodies resulted in increased cross-priming of CD8^+^ T cells by LCs (Klechevsky et al., 2008). We have shown that conjugation of the DC-SIGN binding Lewis-type blood antigens (Le^*b*^ or Le^*X*^) or an antibody recognizing DC-SIGN to liposomes resulted in an enhanced uptake by DC-SIGN^+^ cells *in vitro* using monocyte-derived DCs (Unger et al., 2012). Of note, there was no difference in uptake between the glycan-modified and the anti-DC-SIGN modified liposomes. These findings demonstrate that not all CLRs share the same preference for glycan-modified antigen, and that they can be presented on multivalent carriers or as small, single glycan-modified peptides. In addition, the distinct expression of CLRs by various DC subsets allows specific targeting to the desired DC type using glycan- or antibody-modified vaccine components.

## Functional Consequences of TLR Activation within the Human Skin

Although the targeting of antigens through DC-SIGN and Langerin has already been shown to result in enhanced CD4^+^ and CD8^+^ T cell responses, early work derived from the group of Steinman provided evidence that only in the presence of a potent adjuvant, CLR-mediated DC targeting induced strong cellular immunity *in vivo* instead of generating tolerance (Bonifaz et al., 2002).

During the past years, research has focused on the identification of suitable adjuvants to combine with intradermal skin vaccination strategies (Alving et al., 2012; Schneider et al., 2012; Oosterhoff et al., 2013). We, and others, have shown that, in general, intradermal administration of soluble TLR ligands does not have major effects on DC migration, maturation, and T cell stimulatory capacity, especially not in relation to the effects that TLR ligands show on *in vitro* generated monocyte-derived DCs (Schneider et al., 2012; Oosterhoff et al., 2013). The discrepancy found on DC maturation after TLR stimulation *in vitro* and *in situ* might be caused by specific, local suppression within the skin microenvironment. Consequently, to overcome this suppression, a strong adjuvant should be administered simultaneous with the DC-targeting vaccine. Surprisingly, the effects of Aldara, a FDA-approved immune response modifier skin cream containing 5% of the TLR7 agonist imiquimod, has shown its potential to improve CD8^+^ T cell responses in mice and patients when topically applied on the skin (Zuber et al., 2004; Fenoglio et al., 2013). It will be interesting to determine the maturation effects of the Aldara cream on the population of APC in human skin, and the induction of CD8 T cell responses in comparison to intradermal injection of soluble R838 (imiquimod; TLR7). Selection of the appropriate adjuvant in combination with an anti-tumor vaccine is therefore essential to induce immunity and avoid tolerance. A more detailed knowledge of the different immune responses induced by CLRs and their interplay with TLRs is needed for the improvement of vaccination strategies using CLR ligands. Separate from TLR ligands allowing DC maturation and migration to the draining lymph nodes, also injection of cytokines in the skin as immunostimulators, such as GM-CSF has been investigated (van den Eertwegh et al., 2012; Grotz et al., 2013).

## The Importance of the Route of Vaccine Administration

The specific route of administration is often determined by the type of adjuvant present in the vaccine. In humans, vaccines containing aluminum-based or oil-in-water adjuvants are administered intramuscular (i.m.) or subcutaneously (s.c.) (Figure 1). Intradermal (i.d.) administration of these vaccines likely causes local irritation, induration, skin inflammation, and granuloma formation. The development of novel adjuvants such as synthetic TLR ligands or cytokines facilitates the use of the i.d. route. Vaccination i.d. has shown significant advantages with respect to dose-sparing and immunogenicity in comparison to other routes (e.g., s.c., i.m., i.v.) (Kenney et al., 2004). This is likely due the presence of multiple DC subsets in the skin. Additionally, skin DCs are generally more prone to become immunogenic than, for example, mucosal DCs. However, i.d. vaccination using standard needle and syringes is technically challenging and inaccurate administration of vaccines can even result in adverse side effects. Moreover, vaccination using standard needle and syringe will deliver the vaccine at one spot. It has not been thoroughly investigated whether simultaneous delivery at different/multiple spots leads to superior responses. Simultaneous delivery could be facilitated by the use of microneedle arrays. Microneedle arrays can go into the skin at very low insertion forces and controlled depth, facilitating effective delivery of vaccines.

Indeed, using solid metal microneedles that were coated with an antigen-containing solution it was shown that within 2 h, 50% of DCs that had emigrated out of murine ear explants were antigen positive (del Pilar Martin et al., 2012). Moreover, compared with s.c. vaccination, a single vaccination with influenza-vaccine coated solid metal microneedles induced potent long-lived immunity and improved protection against influenza virus (Koutsonanos et al., 2011). A new generation of microneedles are “reservoir-integrated skin interface devices” that allow microneedle-guided transport of the vaccine while remaining inserted in the skin (van der Maaden et al., 2012). Recently, the microneedle delivery techniques have been broadened by the generation of nanoporous out-of-plane microneedle arrays from ceramic material (Bystrova and Luttge, 2011). The ceramic nanoporous microneedles allow the investigation of a range of parameters related to delivery performance (e.g., cargo loading capacity, amount and arrangement of microneedles, microneedle tip shape). The use of so-called out-of-plane microneedle arrays allows a standardized and regulated delivery of the vaccine to dermal DCs. The intrusion depth into the skin is self-defined by the microneedle length. Notably, this approach also facilitates targeting of the LCs, which is more difficult when using i.d. injection using classic syringe/needles. Moreover, by varying the amount and arrangement of microneedles on the array as well as the microneedle tip shape, different skin DC subset(s) may be triggered. The impact of such microneedle arrays on the efficacy of skin DCs targeting and/or the induction of T-cell mediated immunity has not been fully investigated yet.

As an alternative, polymeric dissolvable microneedle arrays are being explored that release the vaccine into the skin but dissolve within minutes leaving no residual sharps waste. Using these polymeric dissolvable microneedles, 23% of the DCs in draining lymph nodes were loaded with microneedle-applied antigen 72 h later. Unfortunately, this study did not address whether this method was superior in vaccine delivery to dermal DCs than conventional immunization strategies (Zaric et al., 2013). However, Sullivan et al. (2010) showed that vaccination of mice against influenza using these dissolvable microneedles induced enhanced protection compared with conventional i.m. vaccination. Also more recently it was demonstrated that microneedle array skin delivery systems of live adenovirus vaccines in mice resulted in potent CD8^+^ T cell priming through stimulating Langerin^−^ DCs, indicating that, in this study, LCs were not the best in inducing cross-presentation, but dermal DCs (Bachy et al., 2013).

## The Skin Micromilieu: An Immune Suppressive Environment?

The main constituent of the skin is keratinocytes. Similar to gut epithelial cells, keratinocytes can sense pathogens and mediate immune responses to discriminate between harmless commensal organisms and harmful pathogens. Epidermal keratinocytes express several TLRs, located either on the cell surface (TLR1, TLR2, TLR4, TLR5, and TLR6) or in endosomes (TLR3 and TLR9) (Begon et al., 2007; Lebre et al., 2007). In addition, TLR7 expression is induced through the triggering of TLR3 by double-stranded RNA, which makes keratinocytes responsive to TLR7 agonists. TLR expression by keratinocytes might be crucial for promoting skin immune responses, as activation of these receptors on human keratinocytes leads to a predominant Th_1_-type immune response and to the production of type I interferons. In addition to antimicrobial peptides, keratinocytes constitutively secrete, or induced to release, numerous cytokines, including IL-1, IL-6, IL-10, IL-18, and TNFα. Of particular interest with regard to the skin in health and disease is the production of IL-1 by keratinocytes. In healthy skin, keratinocytes constitutively synthesize both pro-IL-1α and pro-IL-1β but cannot process them or secrete them in their active forms. Following exposure to stimuli such as UV irradiation, keratinocytes process and release IL-1β through the activation of the inflammasome. Keratinocytes are also an important source of chemokines and express chemokine receptors, and therefore can modulate an immune response by attracting different cell types into the skin. By expressing CC-chemokine ligand 20 (CCL20), CXC-chemokine ligand 9 (CXCL9), CXCL10, and CXCL11 activated keratinocytes selectively attract effector T cells to the skin during diseases or recruit neutrophils or regulate the trafficking of Langerhans cell precursors to the epithelium.

In contrast, TGFβ is a cytokine with anti-inflammatory properties that is produced by different cell types in the skin, such as LC and keratinocytes. LC-produced TGF-β_1_ has been shown to act in a autocrine/paracrine fashion and to maintain the LC in the epidermis, as inferred from emigration of LC from the skin upon abrogating TGF-β_1_ signaling (Bobr et al., 2012). These data suggest that blocking TGF-β_1_ signaling (via anti-TGFβ-RI Abs or pharmacological inhibitors) might be beneficial to include in vaccines that aim to induce antigen-specific CD8^+^ effector T cells by targeting LC, such as in cancer. The other main producers of TGF-β_1_ in the skin are keratinocytes. It has been shown that TGF-β_1_ levels rise upon wounding of the skin or chronic psoriasis (Flisiak et al., 2002, 2003; Wang et al., 2006), but also premalignant keratinocytes express elevated levels of TGF-β_1_. The increased levels of TGF-β_1_ were associated with enhanced LC migration and significantly affected dermal DC composition: increased numbers of skin DCs and pDCs had emigrated and were detected in skin-draining LN, while the influx of blood-derived pDC and DC precursors into the skin was highly increased.

## Future Directions

A lot of knowledge has been gathered on the presence of different human skin-resident APCs, at distinct locations. The expression of innate receptors such as C-type lectins and TLR has been well characterized, and four subsets have been identified individually on their efficacy to mature, migrate, and cross-present antigen for the induction of CD8^+^ T cells. Based on the expression of different set of TLR and CLR it has been speculated that these APC subsets (pDC and mDC) have a division of labor. Some subsets are crucial in the recognition of soluble antigens, while others play a major role in particulate recognition of glycosylated bacterial or viral products. Although we are beginning to understand the function of these APC subsets individually, we hardly have any knowledge available on the function of these APC *in situ* in the human skin. In particular how the suppressive network of keratinocytes may imprint local APC as well as be involved in the inflammatory activation and trigger APC to mature and migrate to draining lymph node for T cell priming. Future vaccination strategies are therefore important to reveal how we can optimally instruct this skin-resident repertoire of APC *in situ* to overcome the suppressive skin micromilieu, and activate them simultaneously (both mDC and pDC) to induce robust antigen T cells responses.

## Conflict of Interest Statement

The authors declare that the research was conducted in the absence of any commercial or financial relationships that could be construed as a potential conflict of interest.

## Appendix

### Key concepts

#### *In vivo* DC targeting

An interesting alternative to vaccination strategies based on *ex vivo* culturing of DCs. Through *in vivo* DC targeting approaches, vaccines are prepared to specifically reach DCs in their natural micromilieau, and hypothetically benefit from the accumulation of antigens in the most effective APCs, with high migratory capacity and while ensuring proper cross-presentation and DC maturation.

#### The four skin resident APC subsets differ in the expression pattern of CLRs

While CD14^+^ dDCs express DC-SIGN, DEC-205, DCIR, Dectin-1, and MR, CD1a^+^ dDCs can be distinguished based on the expression of MGL and low levels of MR, DEC-205, and DC-SIGN. On the other hand, LCs are the only APCs that express Langerin. LCs also express DEC-205. In contrast, CD141^+^ DCs express DEC-205 and CLEC9A.

#### Advantages on the use of glycans to target CLRs on skin APCs

In contrast to other APC-specific receptors used in *in vivo* DC targeting vaccination strategies, the natural ligands of CLRs are low immunogenic, can be mass-produced by chemical methods, and can be easily conjugated to the antigen of choice.

#### Choice of adjuvant

Several CLRs are able to elicit a signaling response that down-modulates the activatory effect of TLRs. However, when higher doses of adjuvants are provided, the net result of this finely regulated signaling system is activatory. Thus, the selection of the appropriate adjuvant is essential for the success of a CLR-targeting strategy.

#### Choice of administration route

The availability of novel TLR-specific powerful adjuvants and the advances in needle engineering have allowed the development of intradermal vaccination devices that allow lower antigen dosage and higher immunogenicity compared to classic vaccination administration routes.
